# Crop, semi-natural, and water features of the cotton agroecosystem as indicators of risk of infestation of two plant bug (Hemiptera: Miridae) pests

**DOI:** 10.3389/finsc.2024.1496184

**Published:** 2024-11-25

**Authors:** Michael J. Brewer

**Affiliations:** Department of Entomology, Texas A&M AgriLife Research, Corpus Christi, TX, United States

**Keywords:** Creontiades signatus, Pseudatomoscelis seriatus, landscape analysis, GIS, pest monitoring

## Abstract

**Introduction:**

This study considers concepts and tools of landscape ecology and geographic information systems (GIS) to prioritize insect monitoring in large-scale crops, using the cotton agroecosystem of the Texas Gulf Coast and two plant bug species (*Creontiades signatus* Distant and *Pseudatomoscelis seriatus* (Reuter) [Hemiptera: Miridae]) as a case study. The two species differed in host plants and time span as cotton pests.

**Methods:**

*C. signatus* and *P. seriatus* abundance in early growth of cotton were regressed on landscape metrics. Comparisons of three approaches to select landscape variables in stepwise multiple regressions were made across spatial scales and two weeks of insect data extracted from monitoring of 21 cotton fields, years 2010 through 2013.

**Results and discussion:**

The spatial variation of plant bug abundance and the landscape features were substantial, aiding the regression approach. For full stepwise regression models using 18 landscape variables, regression model fit using *C. signatus* data was modestly better in week one of sampling when *C. signatus* adults and young nymphs were detected (*R* ^2^ range of 0.56 to 0.82), as compared with model fit at week two (*R* ^2^ range of 0.49 to 0.77). The smallest scale (2.5 km radius) models had the greatest number of variables selected and highest *R* ^2^, while two broader scales (5 and 10 km) and truncating the models to three variables produced a narrower range of *R* ^2^s (0.49 to 0.62) and more consistent entry of variables. Wetland composition had a consistent positive association with *C. signatus* abundance, supporting its association with seepweeds which are common in coastal wetlands. When selected, the composition of cotton and grassland/shrubland/pasture also had a positive association with *C. signatus* abundance. Aggregation metrics were also relevant, but composition metrics in the models were arguably more easily utilized in prioritizing insect monitoring. In contrast, there were few significant regressions using *P. seriatus* data, possibly due to the widespread distribution of its weedy host plants and lower abundance. Overall, selected landscape features served as indicators of *C. signatus* infestation potential in cotton particularly grown near coastal wetlands, but landscape features were not useful for *P. seriatus* infestation potential in cotton.

## Introduction

Insect monitoring (i.e., field sampling, estimating pest density, and comparing estimates to economic thresholds during crop development) is traditionally used to inform decisions of whether to use insecticides to prevent pest populations from increasing to levels causing economic harm (1, 2). This study considers concepts and tools of landscape ecology and geographic information systems (GIS) to help prioritize insect monitoring in large-scale crops. There is a history of using spatial features of agroecosystems to improve understanding of pest ecology and management. A common spatial consideration is linking pest population increase to temperature gradients based on temperature-dependent insect development and seasonal weather patterns (3). Agroecosystem landscape features may also be linked to pest management, including prediction of pest infestations, natural pest control (4), and insect colonization of crops (5); devising planting strategies to lower pest threat (6); and evaluating the invasion potential of pests (7). These examples operate from local (field/farm level) to regional scales, depending on application and sensitivity to landscape features (3, 6, 8).

The agroecosystem of the Texas Gulf Coast has a mix of crop, semi-natural, and water features that are relevant to the ecology of two plant bug species [*Creontiades signatus* Distant and *Pseudatomoscelis seriatus* (Reuter) (Hemiptera: Miridae)], which are pests of cotton [*Gossypium hirsutum* L. (Malvaceae)]. *Creontiades signatus* was first detected in the cotton agroecosystem in the early 2000s in the Texas Gulf Coast region (9), and *P. seriatus* has been a long-established resident of cotton in the southern US (10). The spatial variation of these species across the cotton agroecosystem is significant (11, 12), justifying the interest in prioritizing insect monitoring efforts where risk is high. They move from non-crop host plants into early season growth of cotton, making timely sampling critical for pest management [i.e., applying insecticides to prevent populations from causing economic harm (11, 13)].

These two plant bug species drive much of the insect pest management activities in cotton along the Texas Gulf Coast from flower bud initiation (squares in cotton terminology) through the first month of fruit set (bolls) in this indeterminate crop. *Creontiades signatus* (common name, verde plant bug) is native to the Gulf coastal region of Texas [United States (US)] and Mexico. It feeds on older squares and young cotton bolls, resulting in damage to lint and seed (14). Its invasiveness in cotton may be linked to some combination of its previous suppression by insecticides that were used to control other pests but are now less frequently used (15) and its spread into cotton planted in the neighborhood of semi-natural lands where *C. signatus* is found. Its host plants include saltwater-tolerant seepweeds (*Saueda* spp.) (Chenopodiaceae) found in coastal wetlands, as well as pigweed (*Amaranthus* spp.) (Amaranthaceae) and London rocket (*Sisymbrium irio* L.) (Brassicaceae) found around cropland and semi-natural areas (9). Adults and nymphs have also been detected in sorghum [*Sorghum bicolor* (L.) Moench (Poaceae)] and soybean [*Glycine max* (L.) Merr. (Fabaceae)] (16).

*Pseudatomoscelis seriatus* (common name, cotton fleahopper) is a long-established resident of cotton in the US Cotton Belt and is considered a pest primarily in Texas and Oklahoma. It feeds on squares and is often considered economically important during the first month of squaring. Resulting square abscission can lead to reduced yield (11). Cotton damage varies spatially, and damage is positively associated with *P. seriatus* population density (10). Factors influencing the pest status of cotton fleahopper include the timing of its movement from weedy hosts to cotton and the cotton development stage when first infestation occurs. Non-crop host plants abundant in South Texas are primarily purple horsemint (*Monarda citriodora* Cerv. ex Lag.) (Lamiaceae), silverleaf nightshade (*Solanum elaeagnifolium* Cav.) (Solanaceae), and woolly croton (*Croton capitatus* Michx.) (Euphorbiaceae) (17).

Monitoring cotton fields at least weekly for *C. signatus* and *P. seriatus* is advisable given the potential for migrating adults to reproduce in cotton. However, time and effort are considerations in sampling insects in cotton in south Texas where 125,000 to 200,000 ha of cotton are grown annually. Economic thresholds and sampling strategies are available but not used in individual fields to the extent advised (i.e., monitoring at least weekly in each field for at least the first 4 weeks of squaring; 11, 13). The concept for prioritizing insect monitoring considered here is that landscape features as indicators of initial plant bug infestation in cotton fields may guide resources allocated for insect monitoring. Geospatial tools may help address two relevant questions: are the spatial arrangements of landscape features of the coastal through inland areas of this region related to plant bug spatial variation in cotton, and are relationships with landscape features similar for *C. signatus* and *P. seriatus* that have similar life history (same insect family) but differ in host plants and time length of their pest status on cotton? The same dilemma and interest in prioritizing monitoring effort spatially are relevant to other pests of large cropping systems where pest monitoring is resource-limited (2, 8).

## Materials and methods

The abundance of *C. signatus* and *P. seriatus* in early growth of cotton was regressed on a suite of landscape metrics. The testable hypothesis was that there was an association of *C. signatus* and *P. seriatus* with landscape metrics. Comparisons of three approaches using stepwise multiple regression were made across several spatial scales. The regressions were done separately for *C. signatus* and *P. seriatus* that differed in their non-crop host plants (source habitat) and time period as a recognized pest of cotton (as one indicator of invasion status).

### Plant bug monitoring in cotton

**Figure 1:**
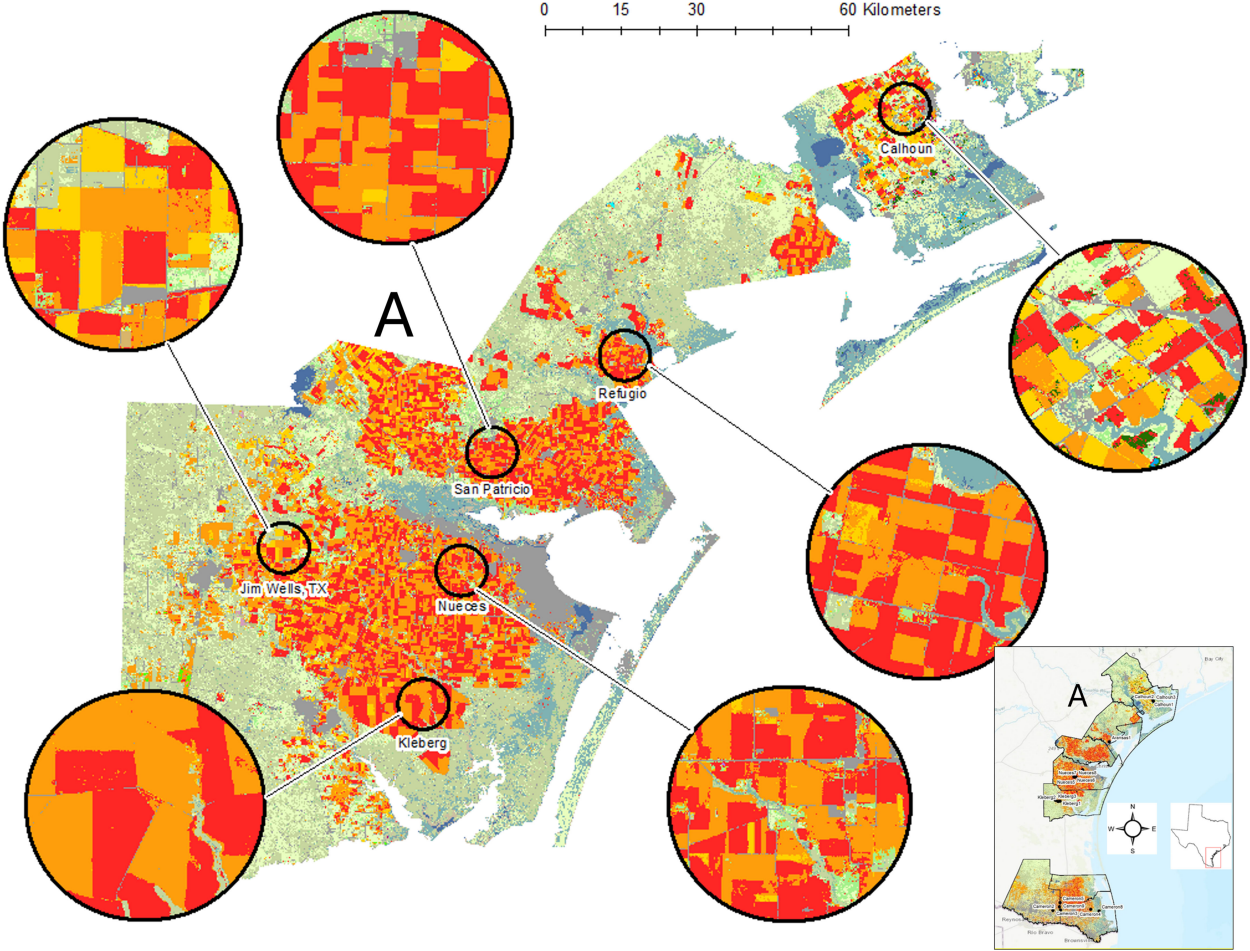
For each year of plant bug sampling, original data layers were extracted from Cropscape (19) and reclassified into 17 new classes focusing on key land cover features such as cotton (red), sorghum (orange), wetlands (blue-green), and grassland/shrubland/pasture (light green). Three spatial scales were used, here showing examples of the 10- km-radius buffers. The full study region is shown as an inset, and the more northern study area of the Texas Gulf Coast (A) is expanded to provide agroecosystem detail.

**Figure 2:**
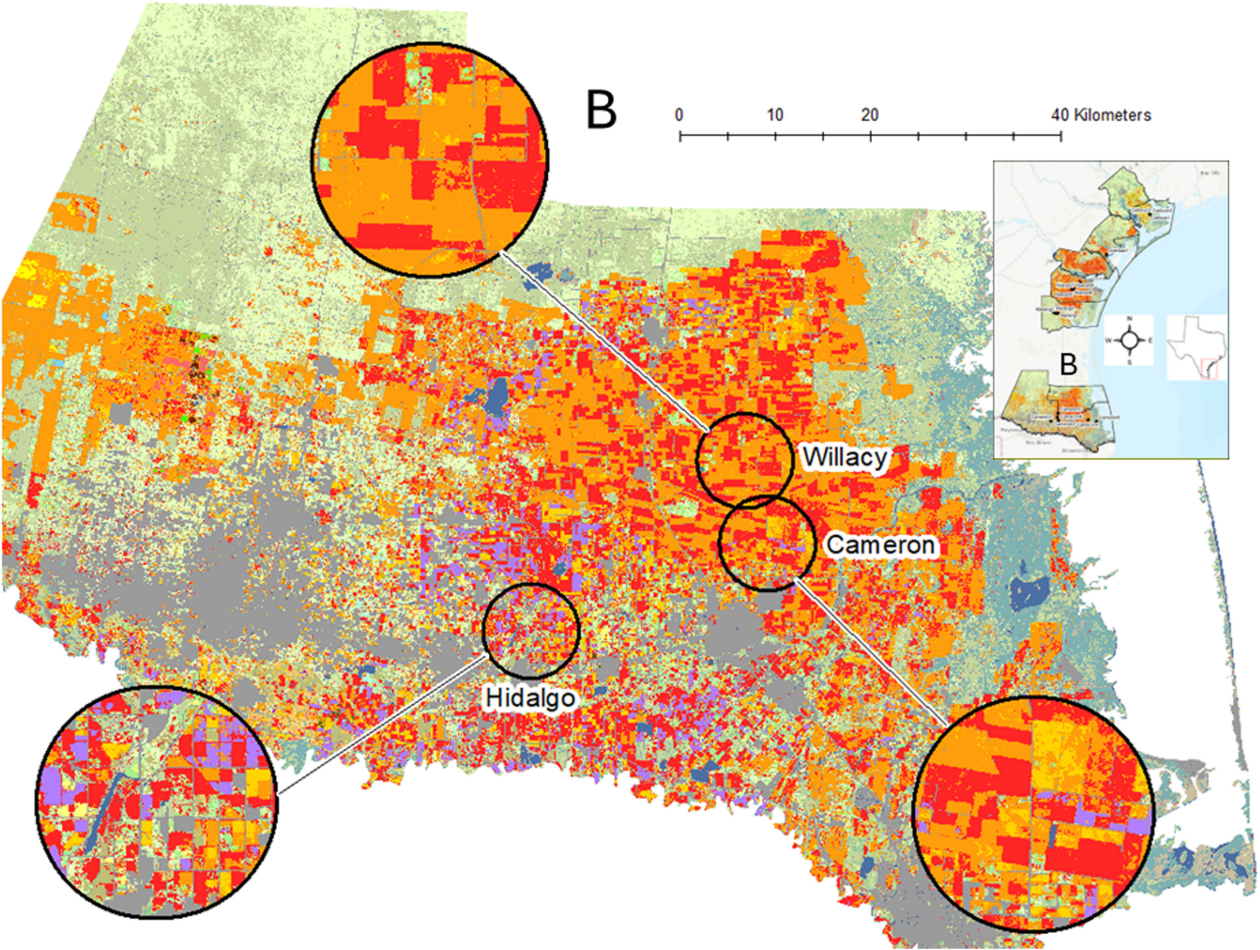
For each year of plant bug sampling, original data layers were extracted from Cropscape (19) and reclassified into 17 new classes focusing on key land cover features such as cotton (red), sorghum (orange), wetlands (blue-green), grassland/shrubland/pasture (light green), and other grass crops such as sugarcane (light purple). Three spatial scales were used, here showing examples of the 10- km-radius buffers. The full study region is shown as an inset, and the more southern study area of the Rio Grande Valley (B) is expanded to provide agroecosystem detail.

Field-specific estimates of *C. signatus* and *P. seriatus* densities were taken from a cotton insect monitoring project, and landscape data were obtained from online data archives. These insects were monitored at 21 commercial cotton fields between 2010 and 2013. The study area was situated within a crop mixture of primarily upland cotton and grain sorghum, a lesser extent of field corn [*Zea mays* L. (Poaceae)], and other crops. These crops were embedded in a large region of semi-natural grassland and shrubland situated along coastlands where wetlands are plentiful and further inland in the lower Texas Gulf Coast including the Rio Grande Valley of Texas, US (**Figures 1**, **2**). Cotton was mostly placed in an annual rotation with sorghum, was primarily rain-fed, and was grown following normal agronomic practices for the region. Cotton field sizes ranged from 200 to 600 ha, and field dimensions varied from irregularly shaped to simple square and rectangular-shaped.

Handheld GPS devices (handhelds) were used to collect insect monitoring data as described in Deleon et al. (12). Briefly, Juno 3B handhelds (Trimble, Sunnyvale, CA) running the application ArcPad [ESRI, Redlands, CA (18)] were loaded with files containing the digitized study area. Data entry fields included number of plants sampled, number of nymphs and adults of *C. signatus* and *P. seriatus* detected, and automated entry of latitude and longitude of each sampling location. After insect sampling, files on the handhelds were transferred to a desktop computer running ArcMap software [ESRI, Redlands, CA (18)]. Data from these files were transferable to other data management and analysis software packages.

*Creontiades signatus* and *P. seriatus* counts were taken in cotton using a beat bucket (13) starting the week of first detection of adults and prior to the use of spray formulations of insecticides to control these or other insects. Each sample site was located about 5 m into randomly selected cotton fields, with a minimum of four sample sites at each of the 21 randomly selected cotton fields. At each sample site, 40 total plants were bent, in groups of four, into the bucket and shaken. This technique dislodged adults and nymphs, which were counted for each plant bug species. Data of each field and date were aggregated to obtain a whole field estimate of *C. signatus* and *P. seriatus* per plant. Adults and early instar nymphs were most of the insects captured during the sampling bout that detected each species. This substantiated that sampling started during the initial phase of annual colonization in cotton. Because variation occurred in plant bug arrival across cotton fields, sampling was conducted weekly over 4 to 6 weeks each year. Two consecutive sampling bouts were used for the analyses for each species after first detection.

### Landscape metrics of the study area

Estimation of landscape metrics was facilitated by overlaying the 21 georeferenced fields onto a cropland data layer. The data layer was a supervised vector-based classification of crops, other land cover types, and water features derived from satellite imagery and available online (Cropscape, 19). Data layers were available for each year of the study as downloads from the Cropscape website. The original data layers extracted from Cropscape were edited with a geoprocessing clip tool to cut the Texas Gulf Coast region that included all study fields for each year of insect sampling, 2010 to 2013 (**Figures 1**, **2**). The original data layers as an aggregate portrayed ca. 90 different crops and other land cover types coded by Cropscape (**Supplementary Table 1**), but principal cropland and other non-crop vegetation were evident by inspection of the color-coded maps generated by Cropscape. Therefore, the original crop data layers were reclassified into 17 new classes. The reclassifications were segmented into crops that were botanically and agriculturally related and other substantial land cover types (e.g., grassland/shrubland/pasture, fallow, and wetlands). Two crops grown in 1 year in the same field (i.e., double cropping) were classified by the summer crop (**Supplementary Table 1**). Open water features were classified as freshwater (e.g., rivers, streams, and lakes) or saltwater (e.g., coastal bays and waterways) and were distinctive from the wetland classification where vegetation occurred.

The landscape metrics were calculated at several spatial scales using circular buffers around the cotton fields sampled, apart from two distance metrics that used the entire study area. Three circular buffers of 2.5, 5, and 10 km radius (= 19.6, 78.5, and 314.3 km^2^, respectively) were created in ArcMap (Create Buffer tool), which extended outward from the centroid of each sampled cotton field (10- km-radius buffer examples shown in **Figures 1**, **2**). The sizes bridged across smaller scales used for less mobile insects and larger scales for more mobile insects (i.e., 20, 21). The centroid of the field was estimated in the GIS using the Feature to Point tool (18). Once completed, they were exported as individual buffer features to simplify geoprocessing. Large bodies of open water (i.e., Gulf of Mexico, bays) and developed areas (i.e., areas of impenetrable surfaces such as clusters of buildings) were occasionally present and were subtracted from the buffer size used in calculating landscape metrics. Using the Extract by Mask tool, files were created that contained the crop data layer within the boundaries of each buffer, and the file format was set to ESRI GRID to import data into a landscape metrics calculation program (Fragstats, 22). ArcMap’s “ ModelBuilder” routine (18) was used to manage the geoprocessing workflow from creating the large reclassified data layer to preparing data for import into Fragstats.

The 17 new classes were available for calculation of landscape metrics, with focus on selected classes relevant to *C. signatus* and *P. seriatus* ecology and pest status in cotton, and dominance in the landscape (see Introduction and **Figures 1**, **2**). The composition and edge metrics were calculated for the classes cotton, sorghum, wetland, grassland/shrubland/pasture, fallow, and corn. Percent composition of landscape [PLD(class)] ranged from 0 to 100 and was computed as the total area (m^2^) of a class in the buffer multiplied by 100 and divided by the area (m^2^) of the buffer. Edge density [ED(class)] had range ≥ 0 and was computed as the sum of edge lengths (m) of a class divided by the total area (m^2^) of the buffer. The aggregation and proximity metrics focused on cotton (the focal crop of this insect monitoring application) and sorghum (the main rotational crop with cotton). Field aggregation was estimated using a clumpiness index [CLP(cotton, sorghum)] with a range from −1 to 1 and patch density [PD(cotton, sorghum)] with range ≥ 0. The proximity metric used was Fragstats’ proximity index [PRX(cotton, sorghum)] with range ≥ 0. The metric CLP(cotton, sorghum) increased in value as patches of the crop became less disaggregated and more clumped. The value of 0 indicated a random distribution of fields. The metric PD(cotton, sorghum) corresponded to the number of patches of the crop in the buffer, adjusted to a per-100 ha basis. The mean proximity index PRX(cotton, sorghum) accounted for both size and proximity of patches of the crop within a buffer. The value of PRX(cotton, sorghum) increased with the number of patches of the crop, and as they became closer and more contiguous in distribution within a buffer. Two distance metrics referred to the nearest distance from a cotton field to freshwater or saltwater features (ENWf and ENWs, respectively, with range ≥ 0). The distance (km) of cotton to the nearest freshwater or saltwater feature was allowed to reach beyond the buffer as needed by accessing the Cropscape clip of the more northern and southern regions of this study (**Figures 1**, **2**). An approximation for the distance calculation was facilitated by converting waterbody polygons to points geopositioned at the approximate centroid of water features using the Feature to Point tool (18). Lines in the GIS representing rivers and streams were unaltered. After the conversion, the nearest distance of the centroid point of a cotton field to the point or line of the nearest water feature was calculated. This procedure reduced computer processing time considerably. A diversity metric, Simpson’s diversity index (SIDI, unitless with range 0 to 1), was calculated using all 17 classes. The SIDI represented the probability that class types of two randomly selected cells of the vector-based classification were different, with the value increasing with increasing number of different classes and with more equal distribution of the area among the classes. More detailed explanations of these metrics are available in Fragstats documentation (22). In total, there were 18 landscape metrics available to the regression procedures (**Supplementary Table 2**). Values of 16 landscape metrics varied by buffer size, while the two distance metrics were independent of buffer size.

### Regression analyses of spatial data

Spatial associations were explored by placing the landscape metrics into a stepwise multiple regression model as explanatory variables for *C. signatus* and *P. seriatus* per plant (23). All 18 metrics were used in a full stepwise regression exercise, followed by two streamlined models that may reduce the number of selected variables further (truncated and cotton-focused stepwise regressions). The modeling exercises were done separately for the two plant bugs and three spatial scales (i.e., buffer sizes). A common (joined) attribute table was created that contained the landscape metrics, insect sampling results, field identifiers, and GPS coordinates for each sampled field. The attribute table was exported from the GIS as an excel file. It was accessed by SAS procedures to generate means and coefficients of variation [CV = 100(standard deviation/mean)] of *C. signatus* and *P. seriatus* per plant across the 21 fields and to run regression exercises (SAS Proc Reg, stepwise option; 24). For the full model regression routines, variables were retained in a forward stepwise process using a 0.15 variable selection (24). From a viewpoint of the principle of parsimony in trend detection (25), *a priori* selections of landscape metrics were based on plant bug ecology and pest status in cotton, and the mix of crops and other landscape features (see Introduction and the preceding section). Yet, the selected metrics from the original 18 may still be prone to high parameterization and less useful and cumbersome for applied applications such as prioritizing insect monitoring spatially. For comparison, two approaches further limited and targeted variable selection. Truncated models used the stepwise regression results but limited the number of selected variables to the three with greatest contribution to model fit. The third approach also used the stepwise process but accessed only the seven landscape variables directly associated with cotton including the diversity metric (cotton-focused models). The Akaike Information Criterion option in SAS was also entertained but selected more variables than the most variable-rich model using the full stepwise model (data not shown). This study emphasized applications of the findings in which few indicator variables may be particularly useful, while more variable-rich approaches may optimize selections more suitable to explore mechanistic interactions (25).

## Results

The heterogeneity of landscape features was indicated across the study region when inspecting means and CVs (**Table 1**). CVs regularly exceeded 10% of the mean (31 of 50 values) with 11 instances exceeding 20%. As a general check on the Cropscape documentation on accuracy (19), ground-truth observations agreed with the classifications of cotton for the 21 sampled fields and of sorghum for a selection of adjacent sorghum fields. *Creontiades signatus* was regularly detected and variable in abundance across cotton fields. Adults per plant using the beat bucket had similar values for the 2 weeks of sampling [mean ~ 0.15 adults per plant (CV ~ 30 for both weeks)]. Densities exceeded the economic threshold of 0.45 bugs per plant (14) in approximately 15% of the fields. In contrast, *P. seriatus* mean abundance was low relative to the economic threshold of 0.15 adults and nymphs per plant (13), but populations were still variable [mean = 0.048 (CV = 25) and 0.0072 (43.1) for the first and second sampling week, respectively]. The overall variability of the data layers and insect counts in cotton was a necessary condition to explore associations of plant bug populations with landscape features of this region.

**Table 1:** Mean (CV) ^a^ of landscape metrics at three spatial scales ^b^ centered on 21 cotton fields along the lower Texas Gulf Coast sampled for the plant bugs *Creontiades signatus* (verde plant bug) and *Pseudatomoscelis seriatus* (cotton fleahopper), 2010–2013.

Class	Metric ^c^	Scale ^b^			
		2.5	5	10	Region
Cotton (Ct)	PLD	26.2 (9.96)	22.6 (9.58)	18.9 (9.32)	
	ED	21.4 (10.3)	20.2 (9.44)	18.4 (9.06)	
	CLP	0.85 (2.53)	0.87 (1.54)	0.87 (1.41)	
	PD	3.16 (12.0)	3.12 (11.6)	3.00 (11.1)	
	PROX	122 (36.8)	54.8 (25.0)	39.9 (18.5)	
	ENWf				6.25 (7.04)
	ENWs				10.1 (16.4)
Sorghum (Sg)	PLD	35.1 (7.29)	32.0 (7.64)	27.5 (7.87)	
	ED	34.3 (1.24)	33.3 (1.16)	30.5 (1.17)	
Wetland (Wt)	PLD	2.68 (29.6)	3.09 (20.1)	4.98 (14.9)	
	ED	11.5 (12.2)	13.4 (12.4)	19.7 (11.0)	
Grassland/Shrubland	PLD	8.40 (16.6)	11.1 (15.7)	12.7 (14.6)	
Pasture (Gr)	ED	29.3 (13.1)	35.4 (10.9)	41.3 (8.11)	
Fallow (Fl)	PLD	3.51 (41.3)	3.86 (37.4)	3.78 (25.2)	
	ED	9.94 (17.5)	10.8 (16.3)	11.8 (12.1)	
Corn (Cn)	PLD	6.52 (27.2)	6.90 (26.1)	5.89 (25.3)	
	ED	10.8 (21.4)	11.4 (18.6)	9.60 (17.0)	
All	SIDI	0.65 (2.72)	0.68 (2.02)	0.71 (1.65)	

a CV (coefficient of variation) = 100 (standard deviation/mean).

b Metrics were derived from circular buffers of 2.5, 5, and 10 km radius (= 19.6, 78.5, and 314.3 km^2^) with the sampled cotton field as the center point. Two distance metrics (ENWf and ENWs) used the entire study region to calculate nearest distance (km) to freshwater and saltwater features.

c Seven metrics calculated for the cotton class, two metrics calculated for five other classes, and one diversity metric calculated across all classes (All). Landscape labels (see text for details): Percent composition of landscape [PLD(6 classes), range 0% to 100%], Edge density [ED(6 classes), range ≥ 0 m per ha], Clumpiness index [CLP(cotton, sorghum), range from −1 to 1, unitless], Patch density [PD(cotton, sorghum), range ≥ 0 count], proximity index [PRX(cotton, sorghum), range ≥ 0, unitless], nearest distance to a fresh or salt waterbody (ENWf and ENWs, respectively, range ≥ 0 km), and Simpson’s diversity index (SIDI, range 0 to 1, unitless).

### Landscape features associated with *Creontiades signatus* early infestation in cotton

All full stepwise regression models (18 landscape metrics available for selection) at the three scales and the two sampling weeks were significant (*p* < 0.0001), with *R* ^2^ values ranging from 0.49 to 0.82 (**Table 2**). Composition metrics were well represented in the models, with composition of one to four classes selected across the models. Wetland composition had a consistent positive association with *C. signatus* abundance in cotton for each model and scale. When selected, composition of cotton and grassland/shrubland/pasture also had a positive association with *C. signatus* abundance in cotton. Clumpiness of cotton and sorghum when selected had a negative association with *C. signatus* abundance. Edge density of the corn class, the diversity metric calculated from all classes, and the cotton distance to fresh or saltwater metric were infrequently selected.

**Table 2:** *Creontiades signatus* (verde plant bug) field average abundance (*C. signatus* per plant) was regressed on landscape metrics calculated at three spatial scales, using three stepwise regression models with different approaches to select landscape metrics.

Week	Scale ^a^	Full model^a^		Truncated model^b^		Cotton-focused model^c^		
		Variable estimates	*R* ^2^, *F*; df; *p*	Variable estimates	*R* ^2^ *F*; df; *p*	Variable estimates	*R* ^2^ *F*; df; *p*
1	2.5	−1.08 + 0.0012 PRX(Sg) + 0.011 PLD(Ct) + 0.034 PLD(Gr) + 0.019 PLD(Wt) + 0.0093 PLD(Fl) – 0.0072 ED(Cn) + 0.99 SIDI	0.82, 17.3; 7, 27; <0.0001	0.021 + 0.00060 PRX(Sg) + 0.017 PLD(Gr) + 0.018 PLD(Wt)	0.61, 16.7; 3, 31; <0.0001	0.37 – 0.0057PLD(Ct) + 0.055 PD(Ct) – 0.011 ENWs	0.40, 7.26; 3, 33; 0.0007
1	5.0	1.98 – 0.0037 PLD(Sg) – 1.87 CLP(Ct) + 0.022 PLD(Wt)	0.56, 14.1; 3, 33; <0.0001	Same		2.86 – 2.76 CLP(Ct) – 0.017 ENWs	0.51, 17.81; 2, 34; <0.0001
1	10.0	1.67 – 1.74 CLP(Sg) + 0.032 PLD(Wt) – 0.082 ENWs	0.62, 18.0; 3, 33; <0.0001	Same		2.27 – 2.89 CLP(Ct) + 0.96 SIDI – 0.015 ENWs	0.57, 14.54; 3, 33; <0.0001
2	2.5	−0.53 + 0.00098 PRX(Sg) + 0.013 PLD(Ct) + 0.039 PLD(Gr) + 0.022 PLD(Wt) + 0011 PLD(Fl) – 0.0054 ED(Cn)	0.77, 16.1; 6, 29; <0.0001	0.054 + 0.00059 PRX(Sg) + 0.015 PLD(Gr) + 0.017 PLD(Wt)	0.55, 13.3; 3, 32; <0.0001	1.72 – 1.54 CLP(Ct) – 0.0094 ENWs	0.37, 10.03; 2, 34; 0.0004
2	5.0	1.56 − 1.54 CLP(Ct) + 0.026 PLD(Wt)	0.49, 16.1; 2, 34; <0.0001	Same		2.67 – 2.56 CLP(Ct) – 0.015 ENWs	0.46, 14.39; 2, 34; <0.0001
2	10.0	1.056 – 1.056 CLP(Ct) + 0.031 PLD(Wt)	0.56, 21.2; 2, 34; <0.0001	Same		2.18 – 2.76 CLP(Ct) + 0.92 SIDI – 0.013 ENWs	0.53, 12.32; 3, 33; <0.0001

21 cotton fields were sampled for *C. signatus* during two consecutive weeks along the Texas Gulf Coast, 2010-2013.

Regression variable labels (see **Table 1** and text for details): Cotton, Ct; Sorghum, Sg; Wetland, Wt; Grassland/Shrubland/Pasture, Gr; Fallow, Fl; Corn, Cn. Percent composition [PLD(6 classes)], Edge density [ED(6 classes)], Clumpiness index [CLP(Ct, Sg)], Patch density [PD(Ct, Sg)], proximity index [PRX(Ct, Sg)], nearest distance to freshwater and saltwater features (ENWf and ENWs, respectively), and Simpson’s diversity index (SIDI).

a Full stepwise regression models with 18 landscape variables available for selection (0.15 selection criteria).

b Truncated models using the first three variables selected by the full stepwise regression models. Same indicates no change from the full model (i.e., variable count was three or less).

c Separate stepwise regressions using seven variables associated with cotton and the diversity metric (0.15 selection criteria).

Considering streamlined models with no greater than three variables that contributed most to explaining variation (truncated models), landscape variables were eliminated in the models at the 2.5-km scale (**Table 2**). The variable reduction resulted in a reduction of the *R* ^2^ (from 0.82 to 0.61 in sampling week 1, and from 0.77 to 0.55 in week 2). Variables selected in the models at 5 and 10 scales were not removed because the full stepwise process using 18 variables resulted in model selection with two or three variables (**Table 2**). The stepwise process using only seven landscape metrics (cotton-focused models) resulted in models retaining two to three variables (**Table 2**). Increasing cotton clumpiness and greater distance to saltwater were associated with lower *C. signatus* abundance, and greater diversity was associated with higher *C. signatus* abundance in two models. The fit of the cotton-focused models (*R* ^2^ values ranged from 0.37 to 0.57) was less than the fit of the truncated models (*R* ^2^ values ranged from 0.49 to 0.62) (**Table 2**).

Overall, regression model fit was modestly better in week 1 of sampling when mostly *C. signatus* adults and young nymphs were detected (*R* ^2^ range of 0.56 to 0.82 for full models), as compared with model fit using week 2 sampling data (*R* ^2^ range of 0.49 to 0.77 for full models). The smallest-scale (2.5 km radius) model had the greatest number of landscape variables selected and highest *R* ^2^. However, truncating the models to three variables produced a narrower range of *R* ^2^ values (0.49 to 0.62) and more consistent entry of landscape variable across the three scales and two sampling weeks compared to the original full models (**Table 2**). The cotton-focused models featured cotton clumpiness and distance to saltwater, which were less utilized in the other two models, but *R* ^2^ values continued to decrease as variable selection was more constrained at the outset of the stepwise process.

### Landscape features associated with *Pseudatomoscelis seriatus* early infestation in cotton

The lower *P. seriatus* populations in cotton may have affected model quality, with only four regressions significant [two full and two truncated models (*p* < 0.05) and no significant cotton-focused models] (**Table 3**). Regressions using data from the second week of sampling (with a greater mix of adults and nymphs) showed improved full model regressions (significant regressions at the 2.5- and 5. 0-km scale with *R* ^2^ > 0.70), compared to those using week 1 sampling data (one significant regression at the 2. 5-km scale with a low *R* ^2^ of 0.36). Furthermore, inconsistencies in the landscape variables selected in the models across scales appeared to be the norm (**Table 3**) compared with the results for *C. signatus* (**Table 2**).

**Table 3:** *Pseudatomoscelis seriatus* (cotton fleahopper) field average abundance (*P. seriatus* per plant) was regressed on landscape metrics calculated at three spatial scales. Two stepwise regression models with different approaches to select landscape metrics are presented.

Week	Scale ^a^	Full model^a^		Truncated model^b^	
		Variable estimates	*R* ^2^, *F*; df; *p*	Variable estimates	*R* ^2^, F; df; *p*
1	2.5	0.15 + 3.69 PLD(Cn) − 0.36 ED(Cn)	0.36, 3.36; 2, 12; 0.069	Same	
1	5.0	– ^c^	–	–	–
1	10.0	–	–	–	–
2	2.5	−0.010 − 0.0042 PLD(Sg) + 0.098 PLD(Wt) − 0.013 ED(Cn) − 0019 ENWf + 0.045 ENWs	0.97, 27.0; 5, 4; 0.0035	−0.062 − 0.0027 PLD(Sg) + 0.057 PLD(Wt) + 0.024 ENWs	0.75, 6.09; 3, 6; 0.030
2	5.0	−0.55 + 0.010 PLD(Ct) + 0.075 PD(Ct) + 0.0068 ED(Gr)	0.74, 6.57; 3, 7; 0.019	Same	
2	10.0	–	–	–	–

21 cotton fields were sampled for *P. seriatus* during two consecutive weeks along the Texas Gulf Coast, 2010-2013.

Regression variable labels (see **Table 1** and text for details): Cotton, Ct; Sorghum, Sg; Wetland, Wt; Grassland/Shrubland/Pasture, Gr; Fallow, Fl; Corn, Cn. Percent composition [PLD(6 classes)], Edge density [ED(6 classes)], Clumpiness index [CLP(Ct, Sg)], Patch density [PD(Ct, Sg)], proximity index [PRX(Ct, Sg)], nearest distance to freshwater and saltwater features (ENWf and ENWs, respectively), and Simpson’s diversity index (SIDI).

a Full models with 18 landscape variables available for selection (0.15 selection criteria).

b Truncated models using the first three variables selected by the full stepwise regression models. Same indicates no change from the full model (i.e., variable count was three or less).

c Selected regressions were not significant (*p* > 0.05, indicated by a dash), including all regressions using only seven variables associated with cotton and the diversity metric (cotton-focused model, data not shown).

## Discussion

In general, the spatial arrangements of the vegetation that comprise a landscape may play a role in determining an organism’s population size. The relationship of pests with their host plants in an agroecosystem is derived from the extent that resources are more or less likely to be present and accessible in spatial mosaics of habitats that differ in landscape structure (26). Thus, the landscape metrics of key features may serve as indicators of pest activity in general, and where to prioritize insect monitoring in the specific application for two plant bug pests of cotton presented here. The landscape relevant to *C. signatus* may be described as coastal wetlands where seepweed hosts are present, as well as grassland/shrubland/pasture and narrow corridors of non-crop lands (field edges of crops) where other selected weedy hosts may occur, such as pigweed and London rocket (9, 16, 27). Both nymphs and adults also have been observed in grain sorghum, which is an important crop grown in rotation with cotton (16). A broader range of non-crop host plants of *P. seriatus* are found from coastal to inland cropping and non-crop areas (17, 27). The primary host plants are woolly croton, silverleaf nightshade, and several species of horsemint (*Monarda* spp.) that occur in the vicinity of cotton from coastal to inland areas of south Texas.

Three approaches to building the regressions and the chosen landscape metrics supported the idea that selected landscape features were associated with early season *C. signatus* abundance in cotton. Because mostly adults and young nymphs were detected the first sampling week, the analyses supported that the main landscape features selected in the regressions (e.g., composition of wetlands) may serve as indicators of *C. signatus* infestation potential in cotton. In contrast, there were few significant regressions using *P. seriatus* data, suggesting little utility in landscape features serving as indicators of *P. seriatus* infestation potential in cotton. It was unfortunate that there was not a robust outcome for both species from the viewpoint of insect monitoring. However, the outcome was not unexpected given the differences in the host plant ranges of the two species. *Creontiades signatus* has strong affiliation with saltwater-tolerant seepweeds found in coastal wetlands (9), which was consistent with the finding that the composition of wetlands was positively associated with *C. signatus.* When only cotton-focused metrics were considered, which eliminated the wetland metric, the nearest distance of cotton to saltwater features was selected. Coastal wetlands fed by saltwater are a common feature along the coastline and bays of the study region (Brewer, personal observation). Aggregation metrics (e.g., clumpiness of cotton) were also relevant, as observed in a study investigating the joint effect of composition and configuration metrics on agroecosystem services (28), but in prioritizing *C. signatus* monitoring across fields, the prominence of composition metrics simplifies understanding of pest risk and strategizing the use of monitoring resources across the large cotton agroecosystem.

The meager association of landscape features with *P. seriatus* abundance in cotton may reveal the broader spatial range of purple horsemint, silverleaf nightshade, and woolly croton that may be found along less maintained crop edges, ditches, and fallow fields from coastal to inland areas (27), making *P. seriatus* a relatively ubiquitous plant bug species spatially (Brewer, personal observation). The 2010 to 2013 study years had highly variable monthly rainfall totals (29, see Corpus Christi and Brownsville regions), which may have hindered the increase of *P. seriatus* populations by stressing its host plants. Increasing *P. seriatus* populations in weedy hosts have been observed in rapidly growing weeds stimulated by above average rainfall from April through July, while lower populations were associated with poor growth during dry droughty periods common to the region (17). In comparison, *C. signatus* also has several host plants throughout the study area but may maintain especially high populations in seepweeds found in coastal wetlands that are less sensitive to droughty conditions (Brewer, personal observation).

Regarding spatial scale, fewer and more consistent variables were selected in the models at the 5 and 10 scales using *C. signatus* data. The greater selection of landscape variables for the full regression model for *C. signatus* at the 2. 5-km scale may indicate specific interactions occurring locally. This was consistent with the viewpoint that fewer landscape variables occurring at higher organizational levels (e.g., composition and configuration of vegetation of an area) may constrain the more complex insect–plant interactions dependent on local conditions captured at finer scales (e.g., selection of more landscape metrics) (30). It is the potential for multi-factor interactions operating at finer scales but conditioned by broader scale influences that makes a solely mechanistic approach to devising pest management recommendations prone to unexpected consequences across a spatially variable large agroecosystem such as cotton production in south Texas.

In contrast, the broader-scale pattern of more consistent landscape variable indicators has utility in regional-level pest management, as opposed to predictors of populations levels in cotton fields used with economic thresholds (1, 2). In cotton, landscape considerations for pest management purposes have been used for cotton planting strategies to reduce *Lygus* (Hemiptera: Miridae) annual infestation of cotton in Arizona, US (6) and reinvasion of boll weevil (*Anthonomus grandis grandis* Boheman) (Coleoptera: Curculionidae) into south Texas, US (7). The findings for *C. signatus* lent support for another application for cotton pest management: prioritizing insect monitoring activities in cotton areas at broad scales, such as a 5- to 10-km radius of fields explored here (equivalent to approximately 19,000 to 78,000 acres or 7,800 to 31,500 hectares). Indeed, the heightened risk of initial infestation of cotton fields in the vicinity of wetlands seen in this study is analogous to the cotton planting recommendations of an Arizona study based on *Lygus*’ affiliation with other selected crops such as alfalfa (6). Such regional pest management considerations in cotton predate spatial analyses and GIS management of insect monitoring data, but landscape ecology concepts paired with GIS tools and spatial analysis provide more objective criteria (e.g., 6, 7, this study) and facilitate data collection and use (12).

Robust findings for both *P. seriatus* and *C. signatus* were not attained. A substantial caveat is that populations levels of the two species differed considerably, as potentially related to weather patterns as noted above. Weather as another spatial feature combined with landscape features has been used by others in evaluating pest activities (3, 31) and may be particularly relevant for *P. seriatus* and its association with weedy hosts that are sensitive to seasonal rainfall (17). Time series analyses using a longer-term data set with insects, weather, and landscape features may improve data quality and interpretation including relationship with weather and possibly shedding light on the invasion status of *C. signatus* (i.e., *C. signatus* is a native species but encroachment into cotton was not seen until the early 2000s, versus *P. seriatus* as a long resident of cotton). Extending insect sampling into non-crop and wetland areas where weedy hosts occur may aid in revealing the relative contributions of weedy host plants as sources of *P. seriatus* and *C. signatus* populations that move to cotton, as well as bolster the case for using selected landscape features as indicators of plant bug infestation potential in cotton. Overall, selected landscape features served as indicators of *C. signatus* infestation potential in cotton particularly grown in the vicinity of coastal wetlands where salt-tolerant host plants were common. In contrast, landscape features were not useful for *P. seriatus* infestation potential on cotton at the levels detected in this study.

## Data Availability

The raw data supporting the conclusions of this article will be made available by the authors, without undue reservation.
