# Supplementary material for: Crop, semi-natural, and water features of the cotton agroecosystem as indicators of risk of infestation of two plant bug (Hemiptera: Miridae) pests
Source: Front Insect Sci. 2024 Nov 25;4:1496184. doi: 10.3389/finsc.2024.1496184 (PMC11625742; doi:10.3389/finsc.2024.1496184)
Supplement: Supplementary file 1 [file Table1.docx]

Supplemental Table 1. The original crop data layers downloaded from Cropscape (19) were reclassified into 17 classes organized into main crops (including double crops categorized by the summer crop), crops botanically and agriculturally related, and other land cover types (e.g., wetland, fallow). The new classes were created based on main vegetative land cover features and relevance to *Creontiades signatus* (verde plant bug) and *Pseudatomoscelis seriatus* (cotton fleahopper) ecology and pest status in cotton.

| Sorghum | Sorghum (4), Dbl Crop WinWht/Sorghum (236) |
| --- | --- |
| Other crop grasses | Rice (3), Barley (21), Rye (27), Oats (28), Millet (29), Speltz (30), Sugarcane (45), Sod/Grass Seed (59), Sugarcane (45) |
| Cotton | Cotton (2)  Dbl Crop WinWht/Cotton (238)  Dbl Crop Soybeans/Cotton (239) |
| Wheat | Durum Wheat (22), Spring Wheat (23),  Winter Wheat (24), Triticale (205) |
| Corn | Corn (1), Sweet Corn (12), Dbl Crop WinWht/Corn (225), Dbl Crop Oats/Corn (226), Dbl Crop Triticale/Corn (228), Dbl Crop Barley/Corn (237),  Dbl Crop Corn/Soybeans (241), Pop Corn (13) |
| Soybean | Soybeans (5), Dbl Crop WinWht/Soybeans (26)  Dbl Crop Soybeans/Oats (240),  Dbl Crop Barley/Soybeans (254) |
| Grassland/Shrubland/Pasture | Other Hay/Non-Alfalfa (37), Clover/Wildflowers (58), Switchgrass (60), Grassland/Pasture (176) |
| Woodland | Deciduous Forest (141), Evergreen Forest (142)  Mixed Forest (143), Shrubland (152),  Christmas Trees (70) |
| Wetland | Herbaceous Wetlands (195), Woody Wetlands (190) |
| Fallow | Fallow/Idle Cropland (61), Barren (131) |
| Legumes | Alfalfa (36), Dry Beans (42), Peas (53), Vetch (224), Peanuts (10) |
| Brassiceae, mustards and sugarbeets | Canola (31), Rape Seed (34), Greens (219)  Cabbage (243), Sugarbeets (41), Radishes (246),  Turnips (247), Cauliflower (244) |
| Solaneacae | Potatoes (43), Peppers (216), Tomato (54) |
| Asteraceae and Convolvulaceae | Sunflower (6), Safflower (33), Sweet Potatoes (46) |
| Cucurbids | Watermelon (48), Cucumbers (50), Cantaloupes (209), Squash (222), Pumpkins (229), Honeydew melons (213) |
| Citrus and fruit trees | Peaches (67), Apples (68), Grapes (69), Citrus (72),  Pecans (74), Apples (68), Grapes (69), Plums (220), Blueberries (242), Olives (211), Oranges (212),  Other Tree Crops (71), Pears (77), Pistachios (204), Cherries (66), Walnuts (76) |
| Other herbaceous, vegetables, fruit and field crops | Other crops (44) , Misc veg/fruit/crop (47), Onion (49), Herbs (57), Carrots (206), Turnips (247), Mint (14), Buckwheat (39) |
| Impervious surfaces ^a^ | Developed/Open Space (121),  Developed/Low Intensity (122)  Developed/Med Intensity (123)  Developed/High Intensity (124) |
| Water (not waterways) ^a^ | Aquaculture (92), Open Water (111) |

Numbers in parentheses are codes used in Cropscape to designate the landscape features (19).

^a^ Large waterbodies and Impervious surfaces were not used in calculating landscape metrics. Their areas were not included as part of the area of the buffer.
