# Supplementary material for: Crop, semi-natural, and water features of the cotton agroecosystem as indicators of risk of infestation of two plant bug (Hemiptera: Miridae) pests
Source: Front Insect Sci. 2024 Nov 25;4:1496184. doi: 10.3389/finsc.2024.1496184 (PMC11625742; doi:10.3389/finsc.2024.1496184)
Supplement: Supplementary file 2 [file Table2.docx]

Supplemental Table 2. Seven metrics calculated for cotton class, two for five other classes, and one diversity metric calculated across all classes (All). An ‘X’ indicates that the variable was calculated and available to the regression procedures.

| Metrics | |  | Class | | | | | | | |
| --- | --- | --- | --- | --- | --- | --- | --- | --- | --- | --- |
| Variable | Type |  | Cotton | Sorghum | Wetland | Grassland/  pasture/  herbaceous | Fallow | Corn | All |  |
| PLD | Composition |  | X | X | X | X | X | X |  |  |
| ED | Edge |  | X | X | X | X | X | X |  |  |
| CLP | Aggregation |  | X |  |  |  |  |  |  |  |
| PD | Aggregation |  | X |  |  |  |  |  |  |  |
| PRX | Proximity |  | X |  |  |  |  |  |  |  |
| ENWf | Distance |  | X |  |  |  |  |  |  |  |
| ENWs | Distance |  | X |  |  |  |  |  |  |  |
| SIDI | Diversity |  |  |  |  |  |  |  | X |  |

Landscape labels (see text for details): Percent composition of landscape (PLD[6 classes], range 0 to 100 percent), Edge density (ED[6 classes], range > 0 m per ha), Clumpiness index (CLP[cotton], range from -1 to 1, unitless), Patch density (PD[cotton], range > 0 count), proximity index (PRX[cotton ], range > 0, unitless), nearest distance to a fresh or salt waterbody (ENWf and ENWs, respectively, range > 0 km), and Simpson's diversity index (SIDI, range 0 to 1, unitless).
